# Crystal structure of tetraaquabis(8-chloro-9,10-dioxo-9,10-dihydroanthracene-1-carboxyl­ato-κ*O* ^1^)cobalt(II) dihydrate

**DOI:** 10.1107/S1600536814020972

**Published:** 2014-09-27

**Authors:** Wen-Juan Cai, Bo Liu, Feng-Yi Liu, Jun-Feng Kou

**Affiliations:** a College of Chemistry and Chemical Engineering, Yunnan Normal University, Kunming 650500, People’s Republic of China

**Keywords:** crystal structure, cobalt, anti­tumor, hydrogen bond

## Abstract

In the title complex, [Co(C_15_H_6_ClO_4_)_2_(H_2_O)_4_]·2H_2_O, the Co^II^ ion is bound by two carboxylate O atoms of two 5-chloro-9,10-anthra­quinone-1-carboxyl­ate anions and four water O atoms in a *trans* conformation, forming an irregular octa­hedral coordination geometry. This arrangement is stabilized by intra­molecular O—H⋯O hydrogen bonds between water and carboxyl­ate. Further O—H⋯O hydrogen bonds between coordinating and non-coordinating water and carboxyl­ate produce layers of mol­ecules that extend parallel to (001). The organic ligands project above and below the plane. Those ligands of adjacent planes are inter­digitated and there are π–π inter­actions between them with centroid–centroid distances of 3.552 (2) and 3.767 (2) Å that generate a three-dimensional supra­molecular structure.

## Related literature   

For the synthesis of the title complex, see: George *et al.* (2006 ▶). The major advantage of metal-based over organic-based drugs is the ability to vary coordination number, geometry and redox states, and metals can also change the pharmacological properties of organic-based drugs by forming coordination complexes with them, see: Hambley (2007 ▶). Anthra­quinones are highly effective chemotherapeutic agents with a wide spectrum of anti­tumor activity, see: Unverferth *et al.* (1983 ▶); Kantrowitz & Bristow (1984 ▶); Stuart *et al.* (1984 ▶); Arcamone (1987 ▶). For related compounds, see: Bruijnincx & Sadler (2008 ▶); Gruber *et al.* (2010 ▶); Neufeind *et al.* (2011 ▶).
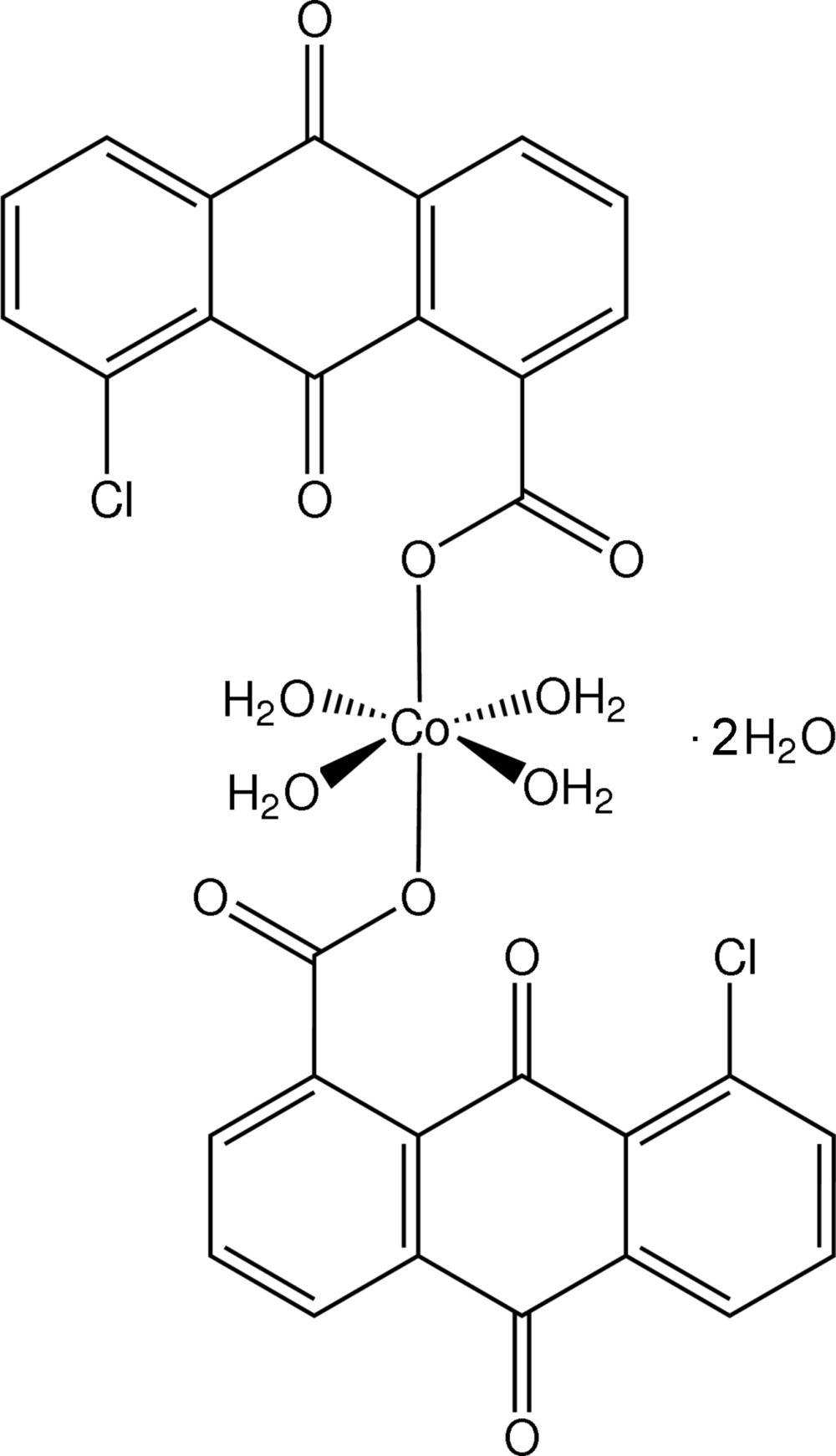


## Experimental   

### Crystal data  

[Co(C_15_H_6_ClO_4_)_2_(H_2_O)_4_]·2H_2_O
*M* *_r_* = 738.32
Triclinic, 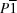

*a* = 6.8655 (14) Å
*b* = 8.1623 (16) Å
*c* = 14.285 (3) Å
α = 73.97 (3)°
β = 88.86 (3)°
γ = 73.35 (3)°
*V* = 735.6 (3) Å^3^
*Z* = 1
Mo *K*α radiation
μ = 0.84 mm^−1^
*T* = 293 K
0.22 × 0.19 × 0.17 mm

### Data collection  

Rigaku MM007-HF CCD (Saturn 724+) diffractometer
Absorption correction: multi-scan (*ABSCOR*; Higashi, 1995 ▶) *T*_min_ = 0.837, *T*_max_ = 0.870
7246 measured reflections
3329 independent reflections
2171 reflections with *I* > 2σ(*I*)
*R*_int_ = 0.041
2 standard reflections every 150 reflections intensity decay: none

### Refinement  

*R*[*F* ^2^ > 2σ(*F* ^2^)] = 0.052
*wR*(*F* ^2^) = 0.179
*S* = 1.12
3329 reflections
235 parameters
9 restraints
H atoms treated by a mixture of independent and constrained refinement
Δρ_max_ = 0.59 e Å^−3^
Δρ_min_ = −0.64 e Å^−3^

### 

Data collection: *CrystalStructure* (Rigaku/MSC, 2006 ▶); cell refinement: *CrystalStructure*; data reduction: *CrystalStructure*; program(s) used to solve structure: *SHELXS97* (Sheldrick, 2008 ▶); program(s) used to refine structure: *SHELXL97* (Sheldrick, 2008 ▶); molecular graphics: *DIAMOND* (Brandenburg, 1999 ▶); software used to prepare material for publication: *SHELXTL* (Sheldrick, 2008 ▶).

## Supplementary Material

Crystal structure: contains datablock(s) I, new_global_publ_block. DOI: 10.1107/S1600536814020972/pj2015sup1.cif

**Fig. 1.**
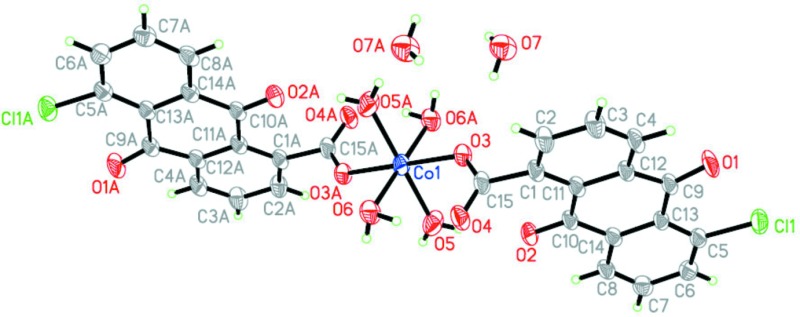
The molecular structure of the title compound, with atom labels and 30 % probability displacement ellipsoids. Symmetry equivalent atoms labelled with an A (eg O1A) are generated by the symmetry operator 1–x, –y, 1–z.

Structure factors: contains datablock(s) I. DOI: 10.1107/S1600536814020972/pj2015Isup2.hkl

x y z . DOI: 10.1107/S1600536814020972/pj2015fig1.tif
The mol­ecular structure of the title compound, with atom labels and 30% probability displacement ellipsoids. Symmetry equivalent atoms labelled with an A (eg O1A) are generated by the symmetry operator 1–*x*, –*y*, 1–*z*.

**Fig. 2.**
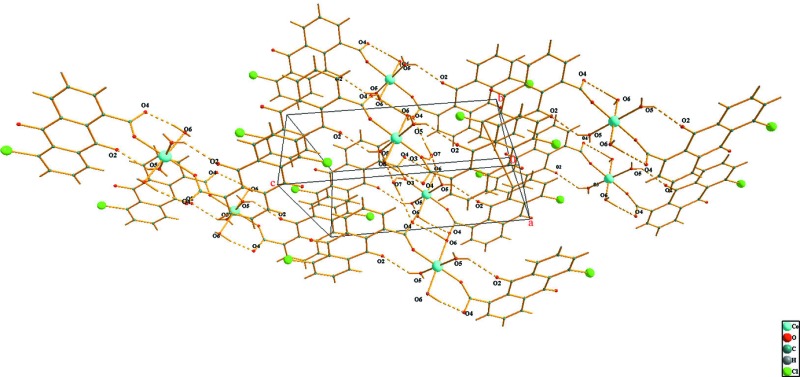
A view of the crystal packing. Hydrogen bonds are shown as brown dashed lines.

. DOI: 10.1107/S1600536814020972/pj2015fig2.tif
A view of the crystal packing. Hydrogen bonds are shown as brown dashed lines.

CCDC reference: 1025297

Additional supporting information:  crystallographic information; 3D view; checkCIF report

## Figures and Tables

**Table 1:** Hydrogen-bond geometry (Å, °)

*D*—H⋯*A*	*D*—H	H⋯*A*	*D*⋯*A*	*D*—H⋯*A*
O7—H7*B*⋯O3	0.80 (4)	2.37 (5)	3.121 (5)	157 (8)
O7—H7*A*⋯O4^i^	0.82 (3)	2.24 (4)	3.049 (4)	169 (8)
O6—H6*B*⋯O4	0.82 (3)	1.92 (3)	2.717 (4)	164 (5)
O6—H6*A*⋯O4^ii^	0.82 (3)	2.17 (4)	2.916 (4)	152 (6)
O5—H5*B*⋯O7^iii^	0.78 (3)	2.08 (4)	2.821 (4)	159 (5)
O5—H5*A*⋯O2	0.81 (3)	2.22 (4)	2.932 (4)	147 (5)

Symmetry codes: (i) 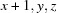; (ii) 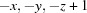; (iii) 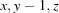.

## supplementary crystallographic information

### S1. Comment

The major advantage of metal-based over organic-based drugs is the ability to vary coordination number, geometry, and redox states and metals can also change the pharmacological properties of organic-based drugs by forming coordination complexes with them. (Hambley *et al.* 2007) Medicinal inorganic chemistry, covering applications of metals in therapeutics and diagnostics, is a field of increasing prominence (Bruijnincx *et al.* 2008) after the discovery and successful clinical applications of the Pt-based anticancer drug cisplatin. Anthraquinones are highly effective chemotherapeutic agents with a wide spectrum of antitumor activity. (Unverferth *et al.* 1983; Kantrowitz *et al.* 1984; Stuart *et al.* 1984; Arcamone *et al.* 1987;). Herein we report the synthesis and structure of the title cobalt(II) anthraquione complex.

The structure of the title complex is shown in Fig. 1, Fig. 2 and hydrogen-bond geometry is given in Table 1. The complex crystallizes in the triclinic space group *P*1 and the asymmetric unit consists of one crystallographically independent co(II) cation, one 5-cyclo-9,10-anthraquinone-1-carboxylate anion, two coordination water molecules and one free water molecule. As shown in Fig.1, the Co(II) ion is bound by two carboxylate O atom (O3,O3A), four water molecules forming an irregular coordination geometry. Strong hydrogen bonds involving an aqua ligand (as a donor) and carboxy O atoms (as an acceptor) may further stabilize the three-dimensional structure (O5···O2=2.932 (4) Å, O5···O7#1=2.821 (4) Å, O6···O4#2=2.916 (4) Å, O6···O4=2.717 (4) Å, symmetry codes:#1 *x*, *y*–1, *z* #2 –*x*, –*y*, –*z*+1). The interstitial water molecules are attached *via* hydrogen bonding to carboxylate O atoms (O7···O4#3=3.049 (4) Å, O7···O3=3.121 (5) Å, symmetry code:#3 *x*+1, *y*, *z*) (Table 1 & Fig.2).

### S2. Experimental

An aqueous solution (2 ml) of cobalt(II) chloride hexahydrate (0.1 mmol, 23.7 mg) was mixed with a methanolic solution (2 mL) of 5-cyclo-9,10-anthraquinone-1-carboxylate (0.1 mmol, 28.6 mg) in presence of two drops of aqueous sodium hydroxide (0.1 *M*). The resulting mixture was allowed to evaporate for one week to yield red crystals, suitable for X-ray work. Yield: 75% (based on the 5-cyclo-9,10-anthraquinone-1-carboxylate)

### S3. Refinement

H atoms attached to carbons were geometrically fixed and allowed to ride on the corresponding non-H atom with C—H = 0.96 Å, and *U*_iso_(H) = 1.2*U*_eq_(C) for other H atoms. For the water molecules, all O—H distances were constrained to be equal within a standard deviation of 0.03Å. Similar H···H distance restraints were applied to restrain the bond angle, but with a larger standard deviation. H atoms of bound water were refined with a single isotropic displacement parameter. Similarly, those of free water were refined with a single, different, U_iso_.

### Crystal data

**Table:**

[Co(C_15_H_6_ClO_4_)_2_(H_2_O)_4_]·2H_2_O	*Z* = 1
*M**_r_* = 738.32	*F*(000) = 377
Triclinic, *P*1	*D*_x_ = 1.667 Mg m^−^^3^
Hall symbol: -P 1	Mo *K*α radiation, λ = 0.71073 Å
*a* = 6.8655 (14) Å	Cell parameters from 25 reflections
*b* = 8.1623 (16) Å	θ = 3.1–25.0°
*c* = 14.285 (3) Å	µ = 0.84 mm^−^^1^
α = 73.97 (3)°	*T* = 293 K
β = 88.86 (3)°	Block, red
γ = 73.35 (3)°	0.22 × 0.19 × 0.17 mm
*V* = 735.6 (3) Å^3^	

### Data collection

**Table:**

Rigaku MM007-HF CCD (Saturn 724+) diffractometer	3329 independent reflections
Radiation source: rotating anode	2171 reflections with *I* > 2σ(*I*)
Confocal monochromator	*R*_int_ = 0.041
ω scans at fixed χ = 45°	θ_max_ = 27.5°, θ_min_ = 3.1°
Absorption correction: multi-scan (*ABSCOR*; Higashi, 1995)	*h* = −7→8
*T*_min_ = 0.837, *T*_max_ = 0.870	*k* = −10→10
7246 measured reflections	*l* = −18→18

### Refinement

**Table:**

Refinement on *F*^2^	Primary atom site location: structure-invariant direct methods
Least-squares matrix: full	Secondary atom site location: difference Fourier map
*R*[*F*^2^ > 2σ(*F*^2^)] = 0.052	Hydrogen site location: inferred from neighbouring sites
*wR*(*F*^2^) = 0.179	H atoms treated by a mixture of independent and constrained refinement
*S* = 1.12	*w* = 1/[σ^2^(*F*_o_^2^) + (0.0859*P*)^2^ + 0.2785*P*] where *P* = (*F*_o_^2^ + 2*F*_c_^2^)/3
3329 reflections	(Δ/σ)_max_ < 0.001
235 parameters	Δρ_max_ = 0.59 e Å^−^^3^
9 restraints	Δρ_min_ = −0.64 e Å^−^^3^

### Special details

**Table:**

Geometry. All e.s.d.'s (except the e.s.d. in the dihedral angle between two l.s. planes) are estimated using the full covariance matrix. The cell e.s.d.'s are taken into account individually in the estimation of e.s.d.'s in distances, angles and torsion angles; correlations between e.s.d.'s in cell parameters are only used when they are defined by crystal symmetry. An approximate (isotropic) treatment of cell e.s.d.'s is used for estimating e.s.d.'s involving l.s. planes.
Refinement. Refinement of *F*^2^ against ALL reflections. The weighted *R*-factor *wR* and goodness of fit *S* are based on *F*^2^, conventional *R*-factors *R* are based on *F*, with *F* set to zero for negative *F*^2^. The threshold expression of *F*^2^ > σ(*F*^2^) is used only for calculating *R*-factors(gt) *etc*. and is not relevant to the choice of reflections for refinement. *R*-factors based on *F*^2^ are statistically about twice as large as those based on *F*, and *R*- factors based on ALL data will be even larger.

### Fractional atomic coordinates and isotropic or equivalent isotropic displacement parameters (Å^2^)

**Table:**

	*x*	*y*	*z*	*U*_iso_*/*U*_eq_	
Co1	0.5000	0.0000	0.5000	0.0521 (3)	
O1	0.2123 (5)	0.3055 (4)	1.0202 (2)	0.0734 (8)	
O2	0.2651 (4)	−0.0771 (3)	0.76542 (19)	0.0618 (7)	
O3	0.3637 (4)	0.1703 (3)	0.58318 (18)	0.0565 (6)	
O4	0.0299 (4)	0.1985 (4)	0.5694 (2)	0.0631 (7)	
O5	0.4564 (6)	−0.2191 (4)	0.6077 (2)	0.0798 (10)	
H5A	0.431 (8)	−0.222 (7)	0.663 (3)	0.101 (9)*	
H5B	0.469 (8)	−0.317 (5)	0.607 (4)	0.101 (9)*	
O6	0.2118 (5)	0.0420 (4)	0.4326 (2)	0.0664 (8)	
H6A	0.181 (8)	−0.050 (6)	0.442 (4)	0.101 (9)*	
H6B	0.141 (7)	0.079 (7)	0.474 (3)	0.101 (9)*	
O7	0.6143 (6)	0.4206 (5)	0.6095 (3)	0.0832 (9)	
H7A	0.724 (7)	0.371 (10)	0.591 (6)	0.16 (2)*	
H7B	0.527 (8)	0.385 (10)	0.594 (6)	0.16 (2)*	
C1	0.1499 (5)	0.3019 (5)	0.6896 (3)	0.0497 (8)	
C2	0.0846 (6)	0.4864 (5)	0.6614 (3)	0.0624 (10)	
H2	0.0601	0.5482	0.5955	0.075*	
C3	0.0552 (7)	0.5804 (5)	0.7312 (3)	0.0666 (11)	
H3	0.0103	0.7043	0.7116	0.080*	
C4	0.0924 (6)	0.4908 (5)	0.8284 (3)	0.0590 (10)	
H4	0.0715	0.5541	0.8746	0.071*	
C5	0.2727 (5)	−0.0750 (5)	1.0984 (3)	0.0506 (8)	
C6	0.3092 (5)	−0.2598 (5)	1.1281 (3)	0.0583 (9)	
H6	0.3266	−0.3212	1.1942	0.070*	
C7	0.3193 (6)	−0.3514 (5)	1.0589 (3)	0.0619 (10)	
H7	0.3390	−0.4738	1.0783	0.074*	
C8	0.3001 (6)	−0.2605 (5)	0.9614 (3)	0.0562 (9)	
H8	0.3107	−0.3230	0.9152	0.067*	
C9	0.2065 (5)	0.2169 (5)	0.9648 (3)	0.0508 (8)	
C10	0.2436 (5)	0.0117 (5)	0.8235 (3)	0.0466 (8)	
C11	0.1858 (5)	0.2093 (5)	0.7891 (3)	0.0476 (8)	
C12	0.1613 (5)	0.3057 (5)	0.8584 (3)	0.0482 (8)	
C13	0.2495 (5)	0.0193 (5)	1.0001 (2)	0.0459 (8)	
C14	0.2651 (5)	−0.0773 (5)	0.9307 (3)	0.0477 (8)	
C15	0.1838 (6)	0.2122 (5)	0.6087 (3)	0.0525 (8)	
Cl1	0.25829 (17)	0.02230 (15)	1.19317 (7)	0.0696 (3)	

### Atomic displacement parameters (Å^2^)

**Table:**

	*U*^11^	*U*^22^	*U*^33^	*U*^12^	*U*^13^	*U*^23^
Co1	0.0629 (5)	0.0479 (4)	0.0531 (4)	−0.0121 (3)	0.0195 (3)	−0.0316 (3)
O1	0.106 (2)	0.0652 (17)	0.0646 (17)	−0.0219 (16)	0.0088 (16)	−0.0472 (14)
O2	0.0817 (18)	0.0561 (14)	0.0632 (16)	−0.0195 (13)	0.0199 (13)	−0.0434 (12)
O3	0.0627 (15)	0.0581 (15)	0.0598 (15)	−0.0146 (12)	0.0214 (12)	−0.0389 (12)
O4	0.0655 (16)	0.0728 (18)	0.0649 (16)	−0.0171 (14)	0.0081 (13)	−0.0453 (14)
O5	0.121 (3)	0.0548 (16)	0.0705 (19)	−0.0255 (17)	0.0424 (19)	−0.0311 (15)
O6	0.0744 (19)	0.0636 (17)	0.0722 (19)	−0.0160 (14)	0.0169 (14)	−0.0419 (15)
O7	0.083 (2)	0.065 (2)	0.104 (3)	−0.0127 (17)	0.012 (2)	−0.0370 (18)
C1	0.0487 (18)	0.0543 (19)	0.055 (2)	−0.0088 (15)	0.0092 (15)	−0.0359 (16)
C2	0.076 (3)	0.054 (2)	0.058 (2)	−0.0064 (19)	0.0075 (19)	−0.0311 (18)
C3	0.082 (3)	0.050 (2)	0.073 (3)	−0.0062 (19)	0.009 (2)	−0.0377 (19)
C4	0.067 (2)	0.056 (2)	0.066 (2)	−0.0103 (18)	0.0129 (19)	−0.0445 (19)
C5	0.0410 (17)	0.065 (2)	0.056 (2)	−0.0157 (16)	0.0121 (15)	−0.0355 (17)
C6	0.054 (2)	0.063 (2)	0.062 (2)	−0.0151 (18)	0.0108 (17)	−0.0269 (18)
C7	0.061 (2)	0.055 (2)	0.075 (3)	−0.0146 (18)	0.0123 (19)	−0.0302 (19)
C8	0.055 (2)	0.058 (2)	0.070 (2)	−0.0168 (17)	0.0136 (18)	−0.0410 (19)
C9	0.0468 (18)	0.058 (2)	0.061 (2)	−0.0128 (15)	0.0140 (16)	−0.0421 (17)
C10	0.0423 (17)	0.0548 (19)	0.057 (2)	−0.0143 (15)	0.0128 (15)	−0.0385 (16)
C11	0.0428 (17)	0.0524 (18)	0.060 (2)	−0.0111 (15)	0.0124 (15)	−0.0387 (16)
C12	0.0446 (17)	0.0546 (19)	0.058 (2)	−0.0133 (15)	0.0136 (15)	−0.0384 (16)
C13	0.0394 (16)	0.0551 (19)	0.0528 (19)	−0.0117 (14)	0.0112 (14)	−0.0332 (16)
C14	0.0404 (16)	0.0548 (19)	0.059 (2)	−0.0126 (15)	0.0102 (15)	−0.0346 (16)
C15	0.065 (2)	0.0494 (19)	0.0489 (19)	−0.0108 (17)	0.0078 (17)	−0.0300 (15)
Cl1	0.0773 (7)	0.0832 (7)	0.0594 (6)	−0.0203 (6)	0.0136 (5)	−0.0419 (5)

### Geometric parameters (Å, º)

**Table:**

Co1—O3^i^	2.083 (2)	C2—H2	0.9300
Co1—O3	2.083 (2)	C3—C4	1.369 (6)
Co1—O5^i^	2.104 (3)	C3—H3	0.9300
Co1—O5	2.104 (3)	C4—C12	1.390 (5)
Co1—O6^i^	2.113 (3)	C4—H4	0.9300
Co1—O6	2.113 (3)	C5—C13	1.390 (5)
O1—C9	1.219 (4)	C5—C6	1.397 (5)
O2—C10	1.225 (4)	C5—Cl1	1.737 (3)
O3—C15	1.259 (4)	C6—C7	1.385 (5)
O4—C15	1.253 (4)	C6—H6	0.9300
O5—H5A	0.81 (3)	C7—C8	1.373 (6)
O5—H5B	0.78 (3)	C7—H7	0.9300
O6—H6A	0.82 (3)	C8—C14	1.387 (5)
O6—H6B	0.82 (3)	C8—H8	0.9300
O7—H7A	0.82 (3)	C9—C12	1.487 (5)
O7—H7B	0.80 (4)	C9—C13	1.492 (5)
C1—C2	1.385 (5)	C10—C11	1.485 (5)
C1—C11	1.402 (5)	C10—C14	1.493 (5)
C1—C15	1.511 (4)	C11—C12	1.405 (4)
C2—C3	1.395 (5)	C13—C14	1.412 (4)
			
O3^i^—Co1—O3	180.00 (10)	C12—C4—H4	119.8
O3^i^—Co1—O5^i^	90.64 (11)	C13—C5—C6	121.2 (3)
O3—Co1—O5^i^	89.36 (11)	C13—C5—Cl1	124.1 (3)
O3^i^—Co1—O5	89.36 (11)	C6—C5—Cl1	114.7 (3)
O3—Co1—O5	90.64 (11)	C7—C6—C5	119.8 (4)
O5^i^—Co1—O5	180.0	C7—C6—H6	120.1
O3^i^—Co1—O6^i^	89.78 (11)	C5—C6—H6	120.1
O3—Co1—O6^i^	90.22 (11)	C8—C7—C6	119.7 (4)
O5^i^—Co1—O6^i^	89.38 (15)	C8—C7—H7	120.1
O5—Co1—O6^i^	90.62 (15)	C6—C7—H7	120.1
O3^i^—Co1—O6	90.22 (11)	C7—C8—C14	121.2 (3)
O3—Co1—O6	89.78 (11)	C7—C8—H8	119.4
O5^i^—Co1—O6	90.62 (15)	C14—C8—H8	119.4
O5—Co1—O6	89.38 (15)	O1—C9—C12	119.7 (3)
O6^i^—Co1—O6	180.0	O1—C9—C13	121.8 (3)
C15—O3—Co1	130.3 (2)	C12—C9—C13	118.4 (3)
Co1—O5—H5A	126 (4)	O2—C10—C11	120.9 (3)
Co1—O5—H5B	131 (4)	O2—C10—C14	120.3 (3)
H5A—O5—H5B	103 (4)	C11—C10—C14	118.7 (3)
Co1—O6—H6A	112 (4)	C1—C11—C12	119.4 (3)
Co1—O6—H6B	98 (4)	C1—C11—C10	121.7 (3)
H6A—O6—H6B	96 (4)	C12—C11—C10	118.9 (3)
H7A—O7—H7B	110 (5)	C4—C12—C11	120.0 (3)
C2—C1—C11	119.5 (3)	C4—C12—C9	117.6 (3)
C2—C1—C15	116.7 (3)	C11—C12—C9	122.4 (3)
C11—C1—C15	123.8 (3)	C5—C13—C14	118.0 (3)
C1—C2—C3	120.6 (4)	C5—C13—C9	123.2 (3)
C1—C2—H2	119.7	C14—C13—C9	118.8 (3)
C3—C2—H2	119.7	C8—C14—C13	120.1 (3)
C4—C3—C2	120.2 (4)	C8—C14—C10	117.9 (3)
C4—C3—H3	119.9	C13—C14—C10	122.1 (3)
C2—C3—H3	119.9	O4—C15—O3	126.6 (3)
C3—C4—C12	120.3 (3)	O4—C15—C1	117.3 (3)
C3—C4—H4	119.8	O3—C15—C1	115.9 (3)
			
O3^i^—Co1—O3—C15	−8 (100)	C13—C9—C12—C4	170.7 (3)
O5^i^—Co1—O3—C15	115.4 (3)	O1—C9—C12—C11	169.1 (3)
O5—Co1—O3—C15	−64.6 (3)	C13—C9—C12—C11	−9.3 (5)
O6^i^—Co1—O3—C15	−155.2 (3)	C6—C5—C13—C14	−0.3 (5)
O6—Co1—O3—C15	24.8 (3)	Cl1—C5—C13—C14	180.0 (2)
C11—C1—C2—C3	0.3 (6)	C6—C5—C13—C9	178.8 (3)
C15—C1—C2—C3	−178.1 (4)	Cl1—C5—C13—C9	−0.9 (5)
C1—C2—C3—C4	0.5 (7)	O1—C9—C13—C5	8.6 (5)
C2—C3—C4—C12	0.5 (6)	C12—C9—C13—C5	−173.1 (3)
C13—C5—C6—C7	−1.1 (6)	O1—C9—C13—C14	−172.2 (3)
Cl1—C5—C6—C7	178.6 (3)	C12—C9—C13—C14	6.1 (5)
C5—C6—C7—C8	2.1 (6)	C7—C8—C14—C13	0.3 (6)
C6—C7—C8—C14	−1.7 (6)	C7—C8—C14—C10	−179.3 (3)
C2—C1—C11—C12	−2.1 (5)	C5—C13—C14—C8	0.8 (5)
C15—C1—C11—C12	176.1 (3)	C9—C13—C14—C8	−178.5 (3)
C2—C1—C11—C10	176.2 (3)	C5—C13—C14—C10	−179.7 (3)
C15—C1—C11—C10	−5.6 (5)	C9—C13—C14—C10	1.1 (5)
O2—C10—C11—C1	1.2 (5)	O2—C10—C14—C8	−3.0 (5)
C14—C10—C11—C1	−175.8 (3)	C11—C10—C14—C8	174.0 (3)
O2—C10—C11—C12	179.5 (3)	O2—C10—C14—C13	177.4 (3)
C14—C10—C11—C12	2.5 (5)	C11—C10—C14—C13	−5.6 (5)
C3—C4—C12—C11	−2.4 (6)	Co1—O3—C15—O4	−20.4 (6)
C3—C4—C12—C9	177.6 (4)	Co1—O3—C15—C1	165.0 (2)
C1—C11—C12—C4	3.2 (5)	C2—C1—C15—O4	−80.0 (5)
C10—C11—C12—C4	−175.2 (3)	C11—C1—C15—O4	101.7 (4)
C1—C11—C12—C9	−176.8 (3)	C2—C1—C15—O3	95.1 (4)
C10—C11—C12—C9	4.8 (5)	C11—C1—C15—O3	−83.2 (4)
O1—C9—C12—C4	−11.0 (5)		

Symmetry code: (i) −*x*+1, −*y*, −*z*+1.

### Hydrogen-bond geometry (Å, º)

**Table:**

*D*—H···*A*	*D*—H	H···*A*	*D*···*A*	*D*—H···*A*
O7—H7*B*···O3	0.80 (4)	2.37 (5)	3.121 (5)	157 (8)
O7—H7*A*···O4^ii^	0.82 (3)	2.24 (4)	3.049 (4)	169 (8)
O6—H6*B*···O4	0.82 (3)	1.92 (3)	2.717 (4)	164 (5)
O6—H6*A*···O4^iii^	0.82 (3)	2.17 (4)	2.916 (4)	152 (6)
O5—H5*B*···O7^iv^	0.78 (3)	2.08 (4)	2.821 (4)	159 (5)
O5—H5*A*···O2	0.81 (3)	2.22 (4)	2.932 (4)	147 (5)

Symmetry codes: (ii) *x*+1, *y*, *z*; (iii) −*x*, −*y*, −*z*+1; (iv) *x*, *y*−1, *z*.
