# Supplementary figures and images for: Crystal structure of tetraaquabis(8-chloro-9,10-dioxo-9,10-dihydroanthracene-1-carboxyl­ato-κO 1)cobalt(II) dihydrate
Source: Acta Crystallogr Sect E Struct Rep Online. 2014 Sep 27;70(Pt 10):m357–8. doi: 10.1107/S1600536814020972 (PMC4257216; doi:10.1107/S1600536814020972)

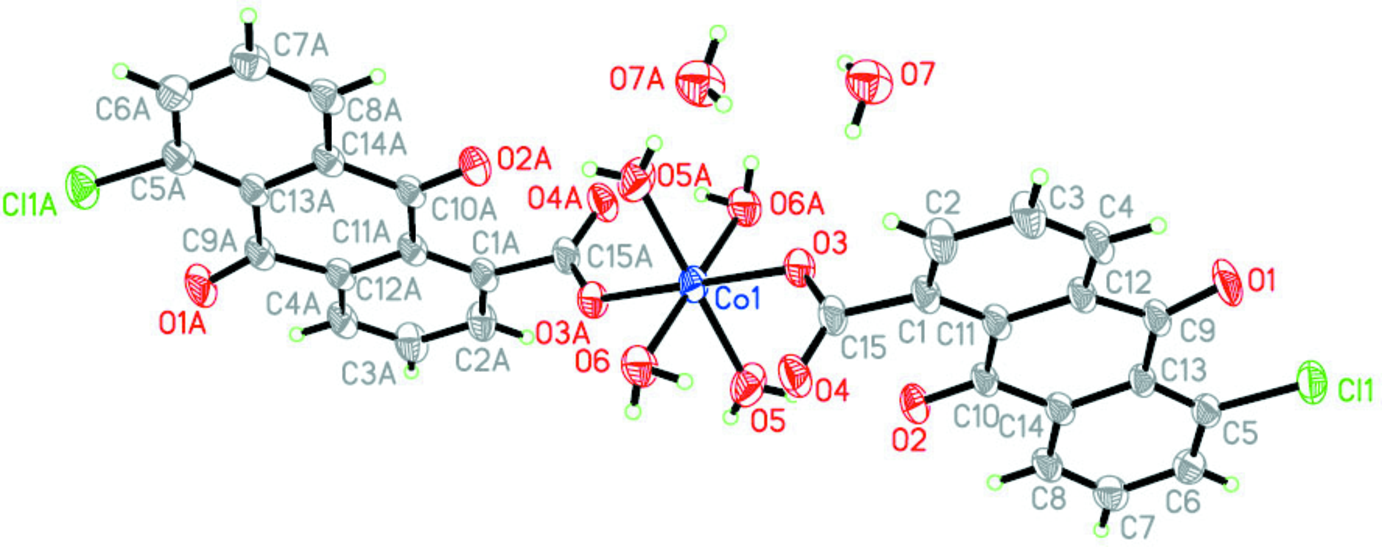

Supplement: Supplementary file 3 [file e-70-0m357-fig1.tif]

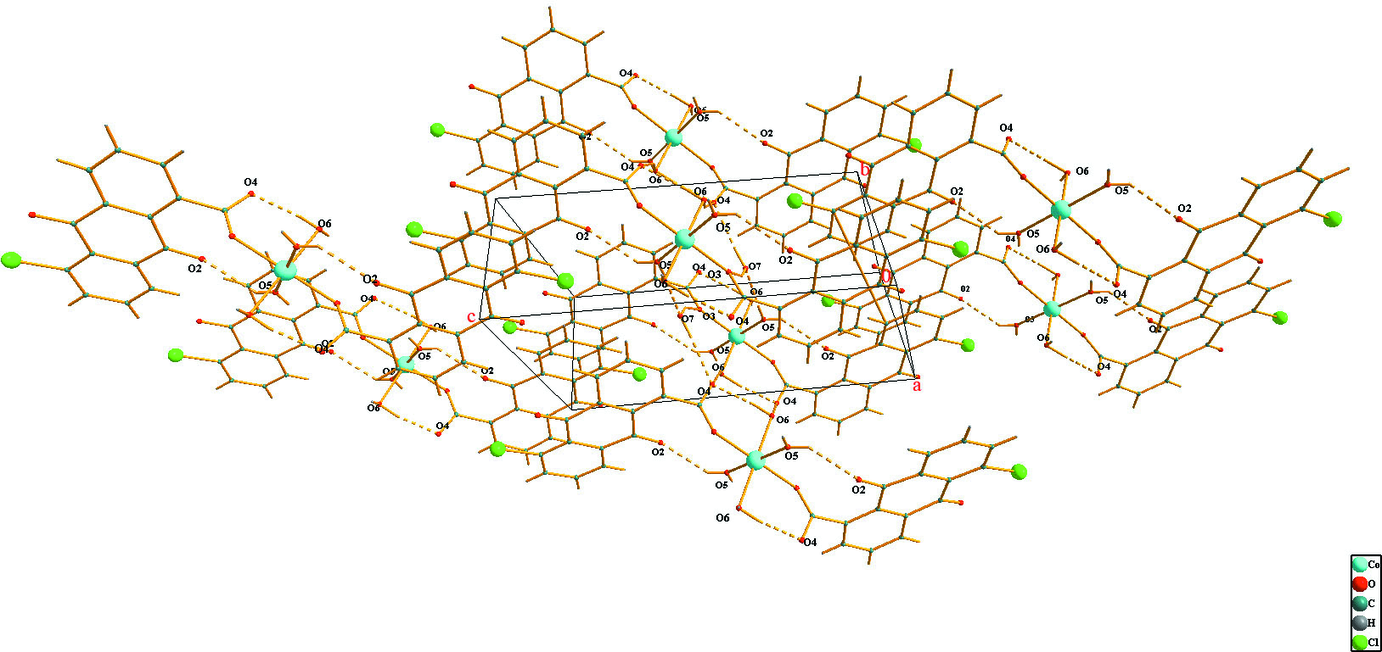

Supplement: Supplementary file 4 [file e-70-0m357-fig2.tif]
